# Durability and Corrosion Properties of Waterborne Coating Systems on Mild Steel Dried under Atmospheric Conditions and by Infrared Radiation

**DOI:** 10.3390/ma15228001

**Published:** 2022-11-12

**Authors:** Ivan Stojanović, Ivan Cindrić, Lovro Turkalj, Marin Kurtela, Daniela Rakela-Ristevski

**Affiliations:** 1 Chair of Materials Protection, Faculty of Mechanical Engineering and Naval Architecture, University of Zagreb, 10002 Zagreb, Croatia; 2 Končar Steel Structures Inc., 10000 Zagreb, Croatia

**Keywords:** corrosion, waterborne coatings, volatile organic compounds (VOCs), infrared (IR) drying

## Abstract

Increasing attention is given to waterborne coatings for corrosion protection due to the lower ecological impact on the environment. It has been found that by using waterborne coatings, the emission of harmful volatile organic compounds (VOCs) is reduced by more than 50 g/L. However, they require longer drying time, their anti-corrosion performance is not as good as solvent-borne coatings and they still have not been developed for all corrosion environments. Another way to reduce VOCs is by using infrared (IR) drying technology. With catalytic infrared radiation, it is possible to cure all surfaces at notably reduced costs compared to traditional systems and in total respect for the environment, thanks to significant energy savings and minimal CO_2_ emissions. The aim of this paper was to evaluate corrosion protective properties of waterborne coatings which were dried with traditional and accelerated drying techniques, i.e., under atmospheric conditions and by using IR technology. Two different coating systems were applied, with and without Zn in the primer. To achieve this goal, the test samples were subjected to electrochemical, corrosion, and physical tests. It was shown that infrared technology does not affect the quality of the coating and it drastically reduces the intercoating interval. A coating system with zinc in the primer showed better overall protection properties after being subjected to impedance and salt spray testing, but generally, solvent-borne coatings still have higher durability than waterborne in extreme marine conditions according to recent research. Microstructure and porosity remained intact and the atomic force microscope confirmed that the flash-off was conducted correctly since there were no pinholes and blisters detected on the coating’s surface. This study can serve as a foundation for further investigations of IC-dried waterborne coatings because there are not many at the moment.

## 1. Introduction

Corrosion in the power transformer industry is of great interest as it can cause serious material and economic damage [1]. It takes an interdisciplinary approach to understand its mechanisms and to find solutions for successful prevention and extension of the machine’s lifetime. Because of carbon steel’s susceptibility to corrosion in an industrial and marine environment, it is important to know its surface properties [2] and to apply various protection methods such as organic coatings [3,4], inhibitors [5,6], and electrochemical metal protection [7,8].

Environmental concern and the devastating impact of global warming has affected all spheres of life, including the coating industry. In that regard, manufacturers are looking for ways to minimize volatile organic compounds (VOCs) by replacing currently used solvent-borne coatings with waterborne [9,10], IR-curable [3], and high-solid coatings [11]. Waterborne coatings still have less practical application than solvent-borne coatings due to the weaker barrier properties they exhibit in highly corrosive environments. To address this problem various additives such as nanofillers [12] and corrosion inhibitors [13,14] for better anti-corrosion performance have been investigated in recent years. Wang et al. [12] used electrochemical impedance spectroscopy (EIS) to examine how the coating resistance changes with the addition of slow-release nanofillers while Gu et al. [13] studied the corrosion rate of graphene-reinforced waterborne coatings using open circuit potential (OCP), EIS, Tafel polarization and salt spray test. Both papers showed that composite coatings exhibit lower corrosion rates and higher impedance modules than pure epoxy coatings. Bastidas et al. [14] also achieved improved corrosion resistance of acrylic waterborne coating by adding microencapsulated corrosion inhibitors which gave higher coating and charge transfer resistance values and lower double-layer capacitance values. Šolić et al. [15] dealt with the influence of different variables in coating preparation and application such as the content of anticorrosive pigment, dry film thickness, and conditioning time. It was observed that pigment content and dry film thickness have the greatest influence and can be adjusted depending on the aggressive environment. High durability can be achieved if the recommended thicknesses are followed according to ISO 12944-5 [16].

Zinc metal particles are also added in coatings to achieve efficient cathodic protection, where zinc acts as a sacrificial anode with a lower potential than the base material. Researchers are aiming to increase the electrical percolation, barrier effect, and galvanic longevity of zinc-rich coatings with various strategies such as surface modification of zinc particles or partial substitution of zinc particles with micro pigments, carbon nanotubes, or graphene [17]. Park et al. [18] modified the surface of zinc particles with the derivatives of phosphoric and phosphonic acid in an aqueous medium. The modification generated a positive effect on corrosion resistance due to the reduced zinc activity and the increased compatibility between the complex layer on the zinc particle and polymer binder matrix. Zhang et al. [19] compared the anti-corrosion performance of waterborne zinc-rich coating with different shapes of zinc particles. Lamellar Zn pigments exhibited better performances than traditional spheric pigments, with the addition that their mass content could be reduced to 25%. Wan et al. [20] showed that adding zinc phosphate can prevent the horizontal diffusion of the corrosive medium into the coating/metal interface and slow down the disbonding of the coating. Both zinc metal particles and zinc phosphate pigment are present in tested waterborne coatings.

Industry can have a positive impact on the environment through innovative and energy-efficient radiation-curing technologies. Thermal energy generated by conventional convection heating takes a long time before it heats the coating and extracts the solvent. out. Alternatively, emitted infrared (IR) waves do not need air as an intermediary, have heating power density six to ten times higher than convective drying, and can penetrate deeper into the coating, reflect from the substrate, and dry the coating from the inside out [21]. The achieved temperatures are much higher with IR curing, which leads to the risk of closing the coating film too quickly, and thus trapping the remaining solvent leaving bubbles on the surface. Radiation curing in terms of UV-curable waterborne coatings has been thoroughly researched in recent years [22,23,24]. However, catalytic IR curing of waterborne coatings has not been thoroughly investigated nor the comparison has been drawn with other drying methods. This research intends to examine the influence of IR radiation on the electrochemical and corrosion properties of waterborne coatings and film formation. Flash-off time is critical when considering these properties because if the film is closed too quickly with heat input from an IR source, surface popping may occur and protection properties will degrade.

In this research, waterborne coatings used for three-layer protection of steel structures in corrosive environments were examined. The goal was to inspect how catalytic IR heating and curing affect the anticorrosive properties and durability of waterborne coatings in comparison with samples dried under atmospheric conditions. For this purpose, coatings were evaluated in an artificial marine environment through a salt spray chamber test followed by a Pull-off adhesion test. Open circuit potential (OCP) and electrochemical impedance spectroscopy (EIS) in 3% NaCl solution were used to obtain the electrochemical potential state and coating resistance, respectively. A detailed study of the test surface quality (roughness and morphology) was executed using atomic force microscopy (AFM) in order to obtain local, three-dimensional, qualitative, and quantitative data and its roughness, using nanometer resolution. Comprehensive microstructural and chemical composition analyses were performed with a scanning electron microscope (SEM) with energy-dispersive X-ray spectroscopy (EDX). Lastly, the comparison between infrared and air-dried samples was given.

## 2. Materials and Methods

**Figure 1:**
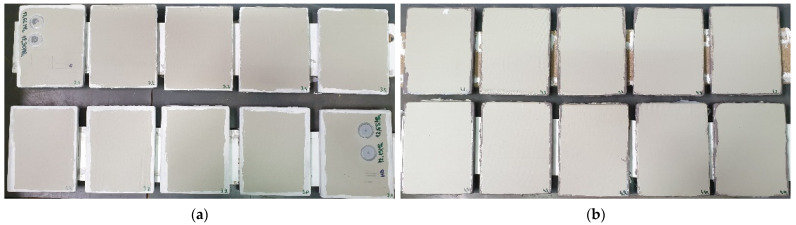
Applied coating system on mild steel plate S235JR + N with (**a**) and without (**b**) zinc in the primer according to Table 2.

Waterborne (WB) two-component (2K) coating systems containing primer, intermediate, and a topcoat layer were evaluated. Infrared radiation was a method used to accelerate crosslinking of the coatings after which they were compared with the samples dried under atmospheric conditions. Required cleanliness Sa 2.5 and a medium degree of roughness, according to ISO 8501-1 [25] and ISO 8503-1 [26], was prepared with steel grit blasting abrasive on the steel test plates (150 × 120 × 8 mm). For each coating used in the research recommended thicknesses, overcoating intervals, gloss, and solids by volume are shown in Table 1. The coating thicknesses were achieved with spiral applicators according to the technical specifications provided by the manufacturer. The drying method, primer type, and manufacturer of applied coating systems are presented in Table 2. Physical and chemical properties of mild steel plates used for coating protection are shown in Table 3 and Table 4. Coatings used in this study are from German manufacturer Chemische Industrie Erlangen GmbH (CHING, Erlangen, Germany) and can be seen applied in Figure 1.

A gas emitter that operates on a principle of flameless catalytic infrared radiation (CIR, Netek IR System A/S, Hobro, Denmark) with medium wavelengths (2–10 μm) was used for accelerated curing of coatings. Gas catalytic IR heaters very efficiently transfer thermal energy that most organic material, including waterborne coatings, easily absorb [27]. The dimensions of the emitter are 600 × 600 mm with a power of 6000 W. Light stroke of a pencil determined whether the coating is sufficiently crosslinked or not. It is considered that fully crosslinked coating has achieved maximum mechanical strength, chemical resistance, thermal stability, adhesion, and other functional properties [28].

Dry film thickness (DFT) measurement was carried out with a non-destructive Elcometer 456 device (Elcometer Limited, Manchester, UK), according to ISO 2808 [29]. Ten measurements were performed on each sample with the device accuracy of ±2.5 µm. According to ISO 4624 [30], an Elcometer 108 Hydraulic Adhesion Tester (Elcometer Limited, Manchester, UK) was used to determine coating adhesion with the device accuracy of ±0.4 MPa. 

**Figure 2:**
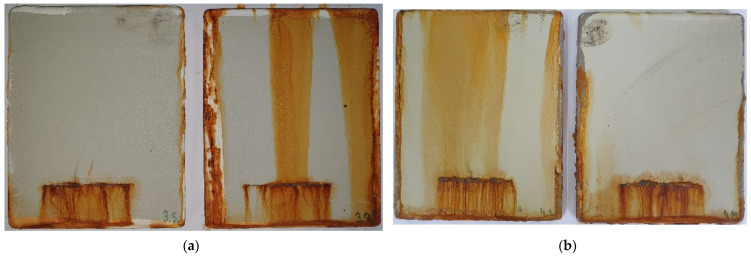
Coating systems with (**a**) and without (**b**) zinc in the primer after 720 h exposure in salt spray chamber according to ISO 9227.

**Figure 3:**
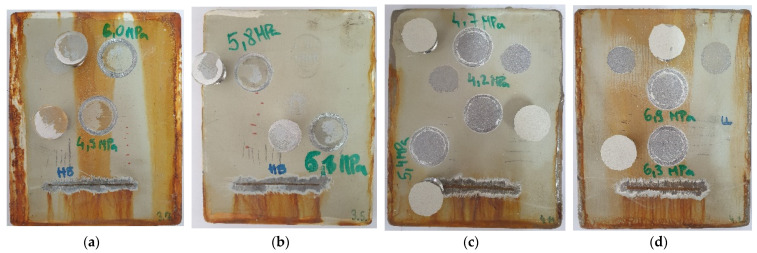
Pull-off testing on coated samples after 720 h of exposure in a salt spray chamber according to ISO 4624. Samples are represented as A1 (**a**), A2 (**b**), B1 (**c**) and B2 (**d**), according to the Table 2.

Corrosion resistance in marine conditions [13,18,19] was examined with an accelerated corrosion laboratory test in the Ascott S450 salt spray chamber (Ascott Analytical Equipment Limited, Staffordshire, UK). The salt chamber temperature was 35 ± 2 °C, the compressed air pressure was 0.7–1.4 bar, and the NaCl solution concentration was 5%, according to ISO 9227 [31]. The coatings degradation was continuously monitored during a period of 720 h after which examination was conducted according to ISO 4628 [32]. Coating systems after 720 h exposure in a salt spray chamber are shown in Figure 2. Upon corrosion testing in the salt spray chamber, pull-off tests were performed on each sample (Figure 3).

Assessment of the coating’s corrosion or stability tendency in sodium chloride solution was conducted through OCP measurement [14]. A saturated calomel electrode acted as a reference electrode [14] in 3% NaCl solution, at room temperature 23 ± 2 °C, against which the open circuit potential of the coating was measured. The duration of OCP measurement was set at 16 ± 1 min.

VersaSTAT 3 Potentiostat/Galvanostat (AMETEK Scientific 131 Instruments, Princeton applied research, Berwyn, PA, USA) was used to evaluate waterborne coating system protection properties via electrochemical impedance spectroscopy. The impedance spectra were obtained after 24 and 720 h in a 3% NaCl solution. The testing was performed at room temperature 23 ± 2 °C. The frequency range was from 10^5^ Hz to 10^−1^ Hz. The working, reference, and auxiliary electrodes formed a three-electrode cell, where the coated sample was the working one, the saturated calomel electrode was the reference, and two graphite sticks were the auxiliary electrodes [14]. The working electrode had a surface of 19.6 cm^2^, while the counter electrodes had a 25.5 cm^2^ surface area. AMETEK ZSimpWin software was used to interpret the data. In order to check the repeatability of the data each measurement was executed in two replications.

FEI Quanta 250 FE Scanning Electron Microscope equipped with an Oxford PENTAFET detector (Oxford Instruments, Belfast, UK) was utilized for the microstructure and the surface morphology scan of IR and air-dried waterborne coating systems. Three different cross-sections were scanned and the representative micrograph is presented in the paper. To analyze the samples, an energy of 20 keV was used.

AFM was used to analyze 3D topography and its roughness using nanometer resolution. Images of samples with dimensions of 20 × 20 μm were taken in contact mode with 256 points in a line (pixel size 78.4 nm), and the examination itself was performed using an AFM device manufactured by Oxford Instruments (Belfast, UK), model MFP-3D Origin.

**Figure 4:**
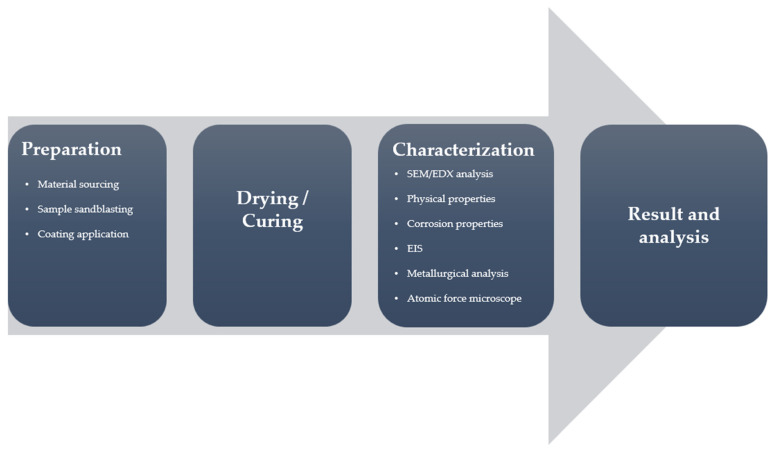
Flow diagram of the methods and procedures used in this study.

Methods and procedures described in this section are visually represented in the flow diagram in Figure 4. 

## 3. Results

### 3.1. Infrared Drying

**Figure 5:**
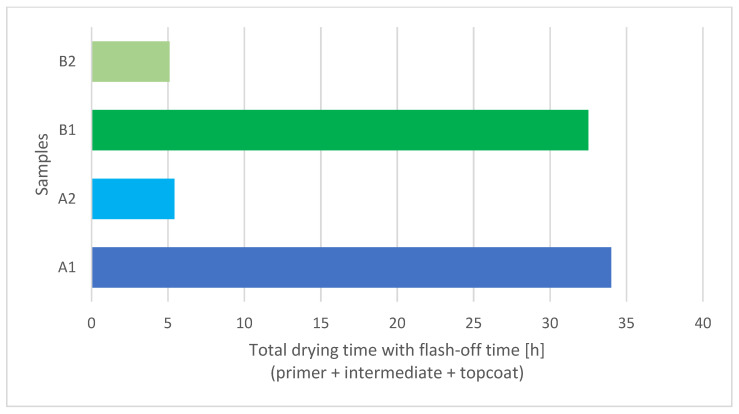
Comparison between drying times of IR (A2, B2) and air-dried (A1, B1) three-layer waterborne coating systems.

In this experiment flash-off time was set at 90 min before every IR curing process. Figure 5 shows a comparison between the total drying times of a waterborne three-layer coating system dried with IR (A2, B2) and under atmospheric conditions (A1, B1). IR drying times were up to six times shorter compared to overall minimum overcoating intervals at 20 °C from Table 1. The difference is even greater according to the manufacturer’s technical specification, which states that the coatings at 20 °C are fully cured after 7 days.

### 3.2. Coating Thickness Measurement

Coated samples did not display significant variations in thickness. ISO 12944-5 [33] specifies that for C5-M the necessary DFT is 200 µm for coating systems with zinc-rich primers (system A) and 240 µm for coating systems without zinc in the primers (system B). The coating thicknesses surpassed the demanded values and within the same system were approximately equal.

### 3.3. Salt Spray Test

Corrosion on the edges was not considered. Coated samples displayed adhesion values between 4.7 and 6.6 MPa, with loss of adhesion between layers for system A, whereas system B completely detached from the substrate. Pull-off values for coating system A were basically the same, while the IR-dried system B sample showed better adhesion properties. Air-dried sample of system B did not pass the adhesion test because the value was below 5 MPa, according to ISO 19244-6 [34]. All samples had shown better adhesion prior to the salt spray test. Table 5 shows the coating thicknesses and the evaluation results of coating protection properties, and adhesion after 720 h testing in the salt spray chamber. Representative samples were chosen for the table since all tested coatings displayed similar corrosion properties in the salt spray chamber.

### 3.4. OCP and EIS Study

Table 6 shows the open circuit potential results after stabilization in a 3% NaCl solution. When exposed to the chloride solution, coating system A showed instability for 720 h, while system B exhibited stable corrosion potential. However, the values were the same within the same coating system.

**Figure 6:**
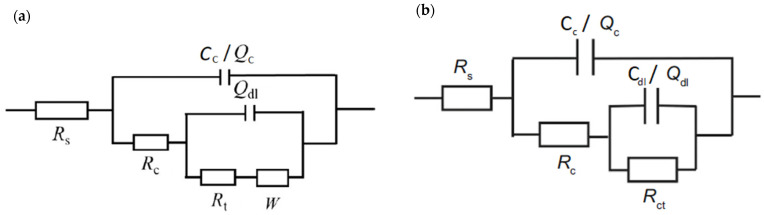
Equivalent electrical circuits used for the fitting of the measured data for (**a**) zinc-rich coating system A and (**b**) coating system B.

To compare corrosion behavior, the coating’s EIS plots were fitted with equivalent circuit models shown in Figure 6. Rs represents the electrolyte resistance, R_c_ is the coating resistance and C_c_ is the coating capacitance. At the steel-electrolyte interface the double-layer capacitance (C_dl_) and charge transfer resistance (R_ct_) are formed. The nonideal capacitance behavior of coating and double-layer is described with constant phase elements Q_c_ and Q_dl_, respectively [35]. The constant phase element (CPE) depends on the empirical constant n, which can range from 0 to 1. CPE acts as a resistor, if *n* = 0, and as a capacitor, if *n* = 1 [14]. Warburg element describes diffusion-controlled reaction happening at a low-frequency band of the impedance spectrum [36]

**Figure 7:**
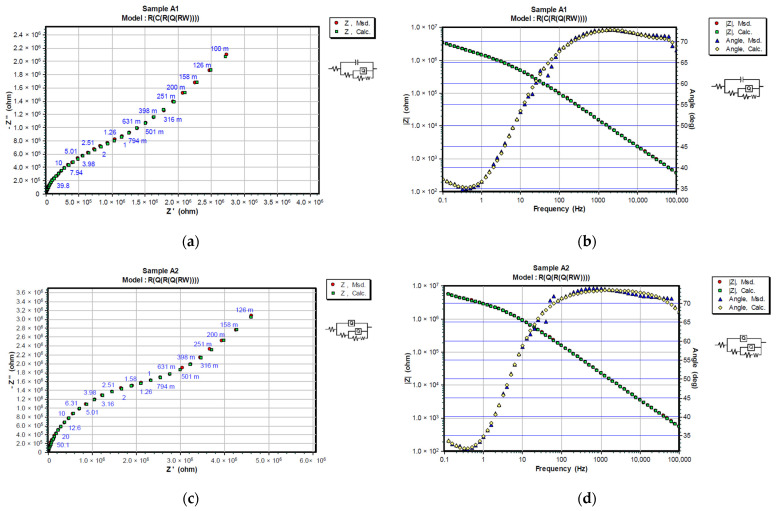

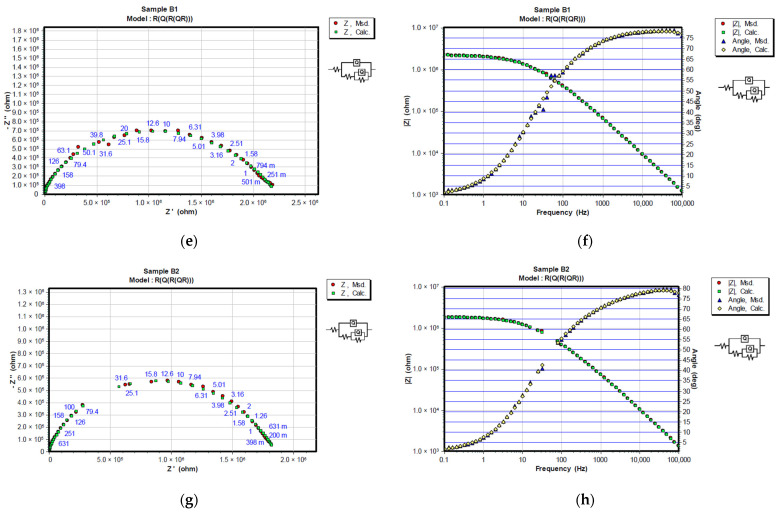
Representation of CNLS simulated and experimental data for each sample after 24 h in 3% NaCl solution with Nyquist and Bode plots. Nyquist diagrams are represented in (**a**) A1, (**c**) A2, (**e**) B1 and (**g**) B2, while Bode and Phase-angle plots are shown in (**b**) A1, (**d**) A2, (**f**) B1 and (**h**) B2.

**Figure 8:**
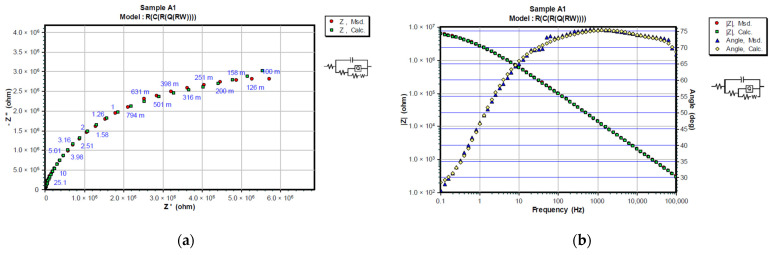

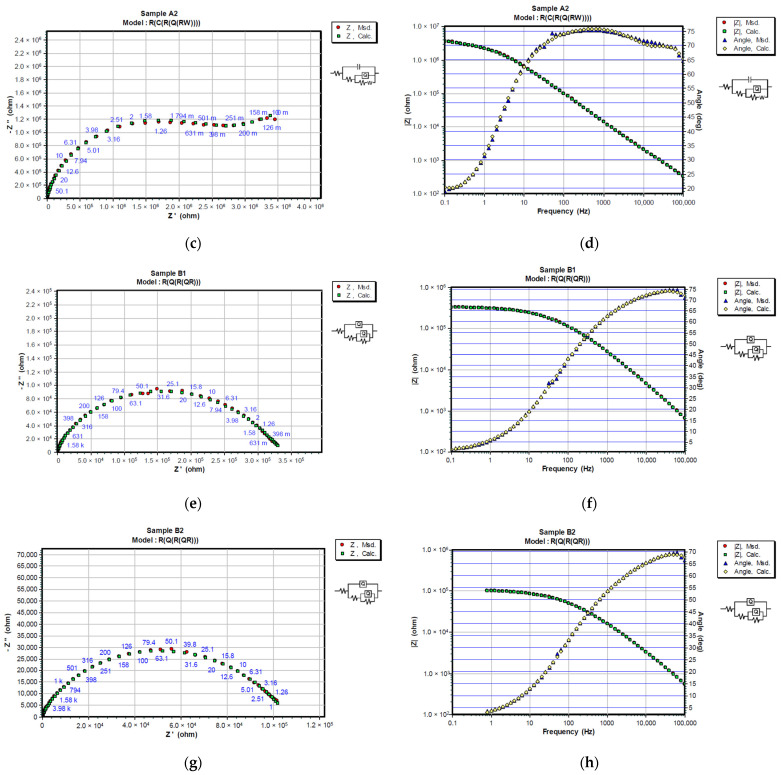
Representation of CNLS simulated and experimental data for each sample after 720 h in 3% NaCl solution with Nyquist and Bode plots. Nyquist diagrams are represented in (**a**) A1, (**c**) A2, (**e**) B1 and (**g**) B2, while Bode and Phase-angle plots are shown in (**b**) A1, (**d**) A2, (**f**) B1 and (**h**) B2.

**Figure 9:**
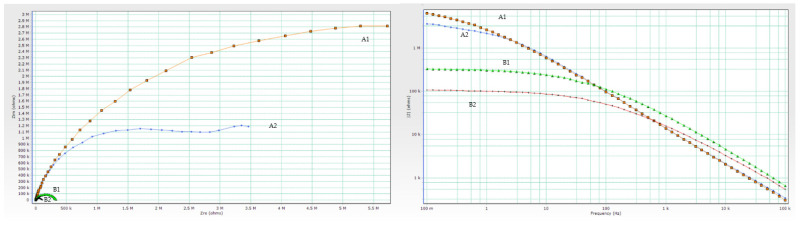
Overlapped Nyquist and Bode plots of coating systems after 720 h immersion in 3% NaCl.

Experimented and simulated data were compared using CNLS (complex non-linear least squares) simulation. Equivalent circuit elements (R_s_, R_c_, R_ct_, Q_c_, and C_dl_) were fitted after 24 and 720 h in 3% NaCl, and their values, together with the values of corresponding χ^2^ modeling errors are shown in Table 7 and Table 8. Only the first measurement is shown since replicated values were in the same range. The chi-square value (χ^2^) method was used to assess the goodness of fit between measured and simulated data [37]. Better fitting results can be attained with lower chi-square values. Nyquist and Bode diagrams in Figure 7 and Figure 8 give a comparison between measured and calculated data after 24 and 720 h in 3% NaCl. To assess the protective performance of the coatings, the impedance modulus at the low frequency was observed. High values of absolute impedance, and high coating resistance directly manifest proper barrier properties [38]. For better comparison, both Nyquist and Bode plots of coating systems after 720 h of immersion in 3% NaCl are overlapped in Figure 9.

### 3.5. SEM and EDX Spectra

**Figure 10:**
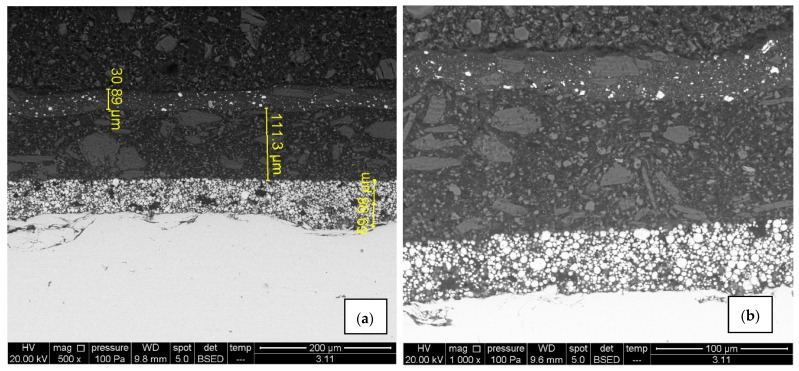

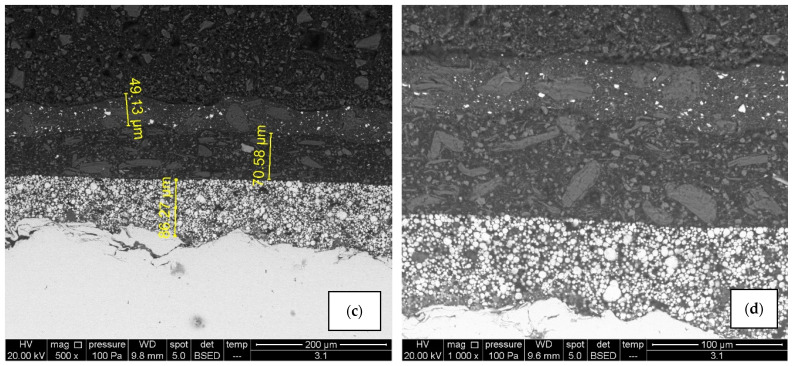
SEM micrographs of air-dried sample A1 (**a**,**b**) and IR- dried sample A2 (**c**,**d**), 500× and 1000× magnification. There are no visible signs of cracks and pores, adhesion is good and zinc is uniformly scattered in the primer.

**Figure 11:**
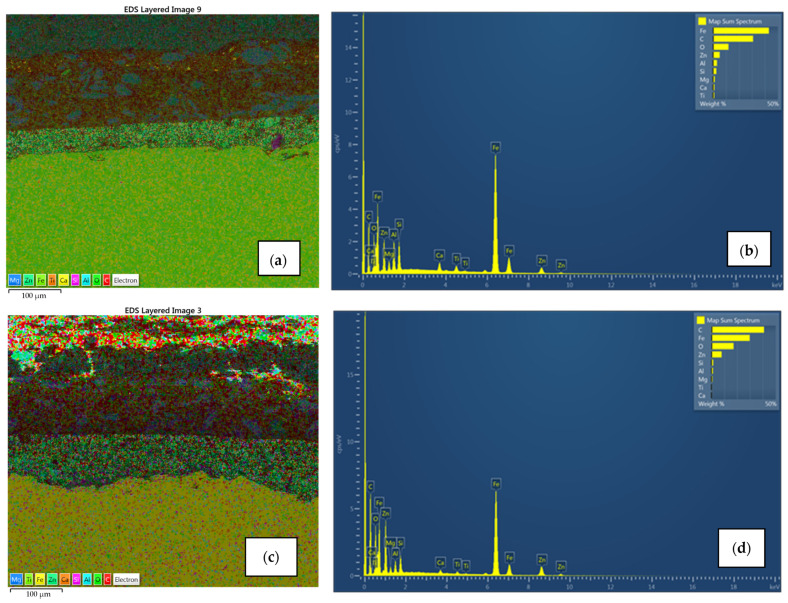
EDX spectroscopy and its elemental mapping for air-dried sample A1 (**a**,**b**) and IR-dried sample A2 (**c**,**d**) with distribution for each element. Zn uniformly scattered in the primer in both cases, Ca spread across the intermediate and topcoat layer with Mg-Si-O inclusions, and topcoat pigmented with Ti.

Surface morphology, pigment distribution, chemical composition, and coating thickness of the coated samples were examined by scanning electron microscope (SEM) with energy-dispersive X-ray spectroscopy. SEM micrograph in Figure 10a,b for sample A1 exhibits no defects (pores, cracks) through the layers, and the coating thickness correlates with the contact measurements. Also, good adhesion of Zn-rich primer to the substrate as well as good adhesion between layers was observed. EDX spectroscopy provided the elemental mapping in which various elements can be seen in each coating layer (Figure 11a,b). Zinc is uniformly scattered across the primer layer, C and O are present in all layers, the topcoat layer is pigmented with Ti, and Ca is distributed across the intermediate and topcoat layers. Mapping also shows Mg-Si-O inclusions evenly spread between two upper layers. All the elements correspond with the product data-sheet. Figure 10c,d show IR-dried sample A2 with no significant difference in adhesion, porosity, and coating thickness when compared to the air-dried sample A1. Cross-section elemental mapping of the IR-dried sample in Figure 11c,b exhibits equivalent elements distribution, meaning IR drying and curing did not negatively influence the distribution of the coatings.

**Figure 12:**
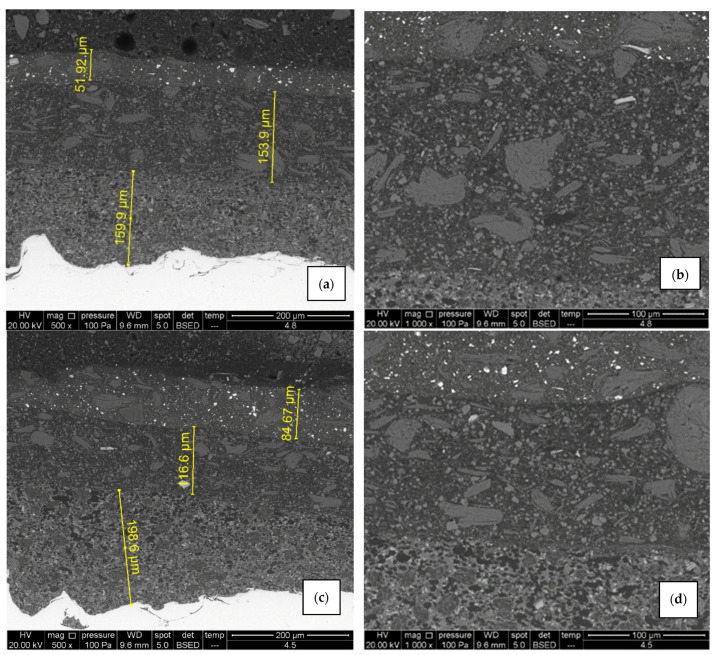
SEM micrographs of air-dried sample B1 (**a**,**b**) and IR- dried sample B2 (**c**,**d**), 500× and 1000× magnification. There are no visible signs of cracks and pores, adhesion is good and elements are evenly distributed in each layer.

**Figure 13:**
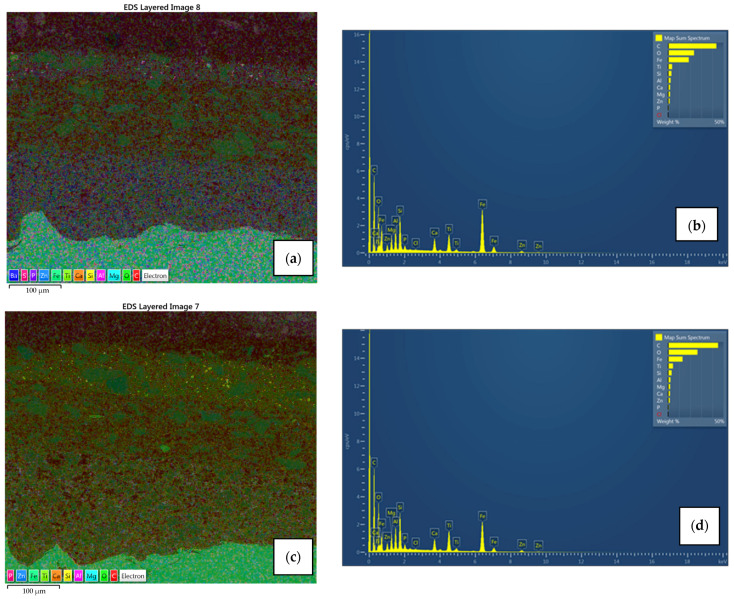
EDX spectroscopy and its elemental mapping for air-dried sample B1 (**a**,**b**) and IR-dried sample B2 (**c**,**d**) with distribution for each element. Ti uniformly scattered both in the primer and in the topcoat, Ca spread across the intermediate layer with Mg-Si-O inclusions, Zn and P in the primer due to zinc-phosphate pigment.

Similar to coating system A, there is no significant difference in microstructure (Figure 12) and chemical composition (Figure 13) between air-dried (sample B1) and IR-dried (sample B2) coating system B. SEM micrograph and elemental mapping display equally good adhesion and element distribution as in the previous case, with the only difference in the primer coat. The pigments in system B primer are titanium dioxide and zinc phosphate instead of zinc dust, meaning Ti, P, Zn, O, and C can be found across the layer. Coating thicknesses correspond with the values obtained with the Elcometer 456 device.

### 3.6. Atomic Force Microscope

**Figure 14:**
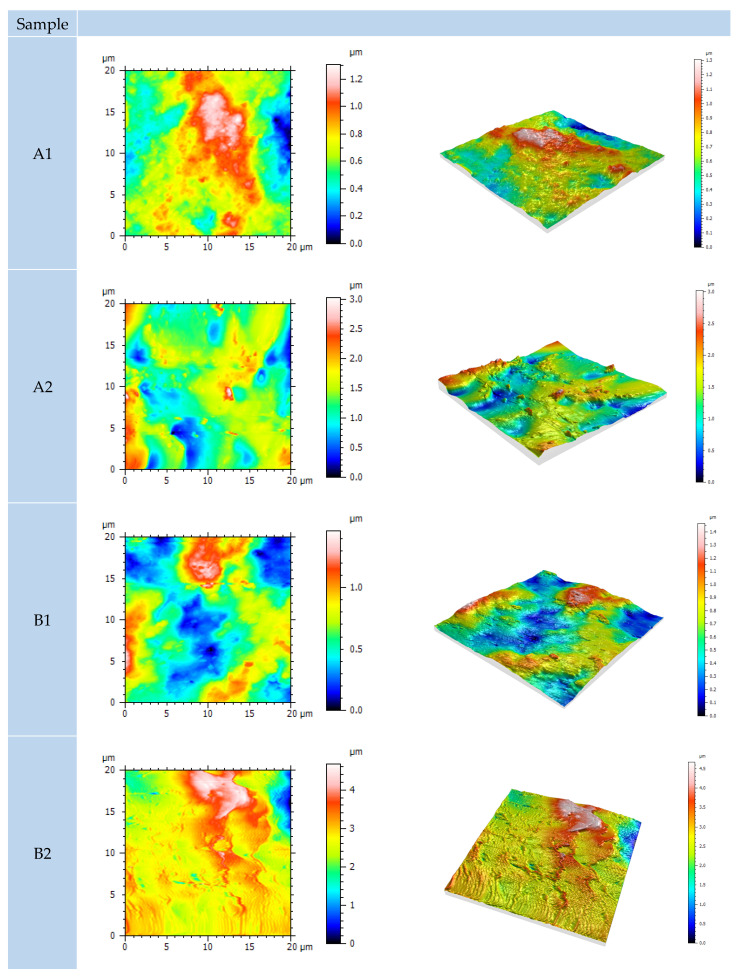
AFM surface topography of differently dried coated samples. Table 2 describes which coating layers are present in samples A1, A2, B1, B2.

In contrast to the previously conducted non-contact tests, using an atomic force microscope (AFM), a detailed scan of the surface layers was carried out, and based on the contact mode of operation, the topography and texture of the surface are defined. The obtained and analyzed results were processed with the program package Mountains SPIP, and presented with the numerical values of the surface parameters in Table 9. The analyzed surfaces of dimensions 20 × 20 μm were scanned at a speed of 1 Hz in a resolution of 512. S_q_ represents the root mean square value of ordinate values within the defined area. It is equivalent to the standard deviation of heights. S_sk_ values represent the degree of bias of the roughness shape (asperity). S_ku_ value is a measure of the sharpness of the roughness profile. S_p_ is the largest peak height value, while S_v_ is the largest pit depth value within the defined area. The sum of those two parameters is defined as S_z_. S_a_ is the extension of R_a_ (arithmetical mean height of a line) to a surface. It expresses the difference in height of each point as an absolute value [37]. In Figure 14, the visual differences in morphology among IR an air-dried sample was observed

## 4. Discussion

It has already been mentioned in the introduction how waterborne coatings positively affect the environment through lesser VOC emissions. One of the significant drawbacks, however, is the surface phenomenon known as popping. Waterborne coatings need a longer flash-off time under atmospheric conditions to dehydrate the coat from 25–50% solids up to 75–90% solids before IR curing. The requirement for dehydration has risen in the last few years since most plants do not have the required space to allow for an ambient temperature flash-off. Too fast dehydration at elevated temperature may trap water under the surface skin during dehydration, which then evaporates during the IR-curing step, causing severe defects and weak properties such as cracking, peeling, pinholes, and blistering [39,40]. In future studies, flash-off time can be reduced by a forced air supply that will remove moisture from the surface [41].

Tested coatings did not exhibit any signs of flacking, rusting, blistering, or cracking according to ISO 4628 [32], after being subjected to 720 h of accelerated corrosion testing in a salt spray chamber according to ISO 9227. The infrared curing process did not have a negative effect on the coating adhesion in a highly corrosive environment. Moreover, the adhesion was improved on system B and went above the required minimum value. Both Hussain et al. [17] and Zhang et al. [19] proved that zinc-rich coatings provide higher corrosion protection with extended durability, especially if they are modified with additional pigments. A zinc-rich coating used in this experiment generally showed better corrosion properties, regardless of the drying method

In the initial phase of the experiment, coated samples showed good barrier properties with polarization resistances higher than 10^6^ Ωcm^2^. Coated samples with zinc in the primer (system A) showed better results during the whole experiment as they preserved their barrier properties over time. The IR-dried sample exhibited higher resistance at the beginning and air-dried at the end of testing. The resistance of the coating changes during exposure due to the penetration of electrolytes into the micropores of the coating. Upon immersion, the polarization resistance (R_p_) can be very high (>10^6^ Ωcm^2^) and usually decreases with the time of exposure to the electrolyte. However, it is not unusual for R_p_ to increase after longer exposure times which is commonly attributed to the formation of zinc corrosion products that block the pores inside the coating [35]. Notably, the diffusion tail was obvious in the impedance spectrum low-frequency band for zinc-rich coating system A. This period of the impedance spectrum was fitted with an equivalent circuit (Figure 6a), where W is the Warburg diffusion impedance [36]. An increase can be seen for sample A1, while sample A2 remained in the same order of magnitude. With no zinc self-healing properties in the primer coating system B experienced a bigger increase in its capacitance and a drop in its resistance values, leading to the probable appearance of defects at the interface of the coating and the steel.

SEM analysis confirmed there were no cracks and pores present in the coatings with good adhesion to the substrate meaning surface preparation was conducted correctly and the coating was applied and dried at the required thickness. This conclusion corresponds with another research conducted on that topic. Šolić et al. [15] emphasize the importance of applying the recommended thickness for proper corrosion protection. Souza dos Santos et al. [42] studied the influence of differently prepared surfaces and concluded abrasive blasting to Sa 2.5 shows better epoxy system adhesion to steel surfaces.

Following the measurement and statistical analysis, higher values of areal topography parameters for IR-dried samples were obtained. In addition, the 3D figures displayed in Figure 13 show a significant difference in the z-axis height value. It is possible that, due to the high-temperature IR curing and fast evaporation of water, lesser homogenization and overflow of the coating were achieved. This may cause microporosity in the film reflecting increased topographic irregularities of the coating surface. The pinholes or blisters were not detected meaning that flash-off time was sufficient prior to IR curing.

## 5. Conclusions

The results of this study showed that IR catalytic drying compared to the existing air-drying technology shortens waterborne coating system drying times while maintaining the same protection properties, which can in return bring great savings in time and energy. The following conclusions can be drawn from this study:

- The intercoating interval between each layer is significantly reduced by catalytic IR drying technology which allows for the coating protection process to be faster.
- Both IR and air-dried zinc-rich coating systems demonstrated good adhesion to the mild steel and exhibited no degradation after salt spray chamber testing.
- SEM analysis showed no cracks and pores in the coatings without detachment from the substrate for both drying methods.
- The EIS results show that coating systems have good barrier properties at the initial phase of immersion. Polarization resistance remained stable for the zinc-enriched coatings system, while the zinc-free system experienced a drop in values. This drop may be associated with the appearance of defects at the steel-coating interface.
- Increased topographic irregularities of the coating surface are observed with IR drying which is probably caused by fast evaporation and quick formation of the film surface.

## Figures and Tables

**Table 1:** Types of coating.

Coating Layers	Gloss	Solids [Vol.%]	Recommended Thickness [µm]	Minimum Overcoating Interval at 20 °C [h]
Primer: Zn (R)	Mat	60	60–80	4
Primer: Epoxy	Mat	49	60–80	2.5
Intermediate: Epoxy	Flat	52	60–130	6
Topcoat: PUR	Mat	46	40–60	24

**Table 2:** Steel test plates with regard to the primer type, drying method, and manufacturer.

Manufacturer	Samples	Coating Systems (Primer–Intermediate–Topcoat)	Drying Method
CHING	A1	Zn(R) EP-EP-PUR	Atm.
	A2	Zn(R) EP-EP-PUR	Infrared
	B1	EP-EP-PUR	Atm.
	B2	EP-EP-PUR	Infrared

**Table 3:** Specification and physical properties of S235JR + N mild steel plates used in this study according to EN 10025/2-2004.

Product	Steel Grade	Norm	Ultimate Tensile Strenght (UTS)	Yield Tensile Strenght (YTS)	L0 [mm]	Elongation [%]
Hot rolled heavy plates	S235JR + N	EN 10025/2-2004	468 MPa	373 MPa	80	30

**Table 4:** Chemical composition of S235JR + N mild steel plates used in this study according to norm EN 10025/2-2004.

C	Si	Mn	P	S	Al	Ti	V	Cu	Ni	Cr	Mo	Nb	B
0.11	0.22	0.80	0.018	0.006	0.037	0.002	0.003	0.04	0.02	0.02	0.002	0.001	0.0002

**Table 5:** Corrosion properties after 30 days (720 h) of testing in the salt spray chamber.

Samples	DFT_mean_	σ_DFT_	Rusting	Cracking	Flaking	Blistering	Pull off [MPa]
	[µm]		ISO 4628-3	ISO 4628-4	ISO 4628-5	ISO 4628-2	
A1	216 (±2.5)	21.8	Ri 0	0(S0)	0(S0)	0(S0)	6.0 (±0.4)
A2	211 (±2.5)	22.7	Ri 0	0(S0)	0(S0)	0(S0)	5.95 (±0.4)
B1	275 (±2.5)	19.4	Ri 0	0(S0)	0(S0)	0(S0)	4.77 (±0.4)
B2	283 (±2.5)	19.9	Ri 0	0(S0)	0(S0)	0(S0)	6.6 (±0.4)

**Table 6:** The open circuit potential results after stabilization in 3% NaCl solution.

Samples	DFT_mean_ [µm]	σ_DFT_	E_corr_ vs. SCE (V)	
			24 h	720 h
A1	202 (±2.5)	12.7	−0.183	−0.458
A2	207 (±2.5)	10.9	−0.142	−0.559
B1	244 (±2.5)	12.3	−0.237	−0.185
B2	239 (±2.5)	17.6	−0.228	−0.198

**Table 7:** Fitted values of equivalent circuit elements (R_s_, C_C_/Q_c_, R_c_, C_dl_/Q_dl_, R_ct,_ W) and the corresponding chi-square value (χ^2^) after 24 h in 3% NaCl.

Samples	R_S_, (10^2^ Ωcm^2^)	n_c_	C_C_/CPE_C_, (10^−8^ Fcm^2^)	R_C_, (10^5^ Ωcm^2^)	n_dl_	C_dl_/CPE_dl_, (10^−7^ Fcm^2^)	R_ct_, (10^6^ Ωcm^2^)	W (10^−7^ Ωcm^2^s^1/2^)	*χ^2^*
A1	0.791	-	42.72	0.0159	0.73	70.19	1.348	4.26	2.45 × 10^−4^
A2	0.622	0.822	3.202	30.46	0.772	1.185	0.908	2.25	5.11 × 10^−4^
B1	0.405	0.881	0.6282	4.142	0.534	0.3594	1.833	-	6.39 × 10^−4^
B2	1.195	0.989	0.1181	0.1768	0.648	0.2483	1.854	-	2.23 × 10^−4^

**Table 8:** Fitted values of equivalent circuit elements (R_s_, C_c_/Q_c_, R_c_, Q_dl_, R_ct_, W) and the corresponding chi-square value (χ^2^) after 720 h xin 3% NaCl.

Samples	R_S_, (10^1^ Ωcm^2^)	n_c_	C_C_/CPE_C_, (10^−8^ Fcm^2^)	R_C_, (10^3^ Ωcm^2^)	n_dl_	CPE_dl_, (10^−7^ Fcm^2^)	R_ct_, (10^6^ Ωcm^2^)	W (10^−7^ Ωcm^2^s^1/2^)	χ^2^
A1	7.615	-	53.22	1.756	0.743	60.35	5.529	4.31	2.78 × 10^−4^
A2	10.41	-	50.43	2.289	0.785	43.77	2.785	4.31	4.00 × 10^−4^
B1	8.806	0.934	0.5657	3.169	0.549	1.503	0.3352	-	1.34 × 10^−4^
B2	7.556	0.881	1.425	4.818	0.535	3.378	0.1033	-	1.19 × 10^−4^

**Table 9:** Results of areal topography parameters.

Samples	S_q_ (µm)	S_sk_	S_ku_	S_p_(µm)	S_v_(µm)	S_z_(µm)	S_a_(µm)
A1	0.2241	0.1042	2.7713	0.6201	0.6873	1.3074	0.1794
A2	0.3898	0.0691	3.2661	1.6982	1.3255	3.0238	0.3055
B1	0.2666	0.3898	2.5259	0.8585	0.6045	1.4631	0.2195
B2	1.1361	1.4912	7.3309	7.0392	2.6734	9.7131	0.8376

## Data Availability

Not applicable.
